# Modern Sociology

**Published:** 1898-11-12

**Authors:** 


### Modern Sociology.

### THE CHILDREN OF CRIMINALS.

By an Official.

Viewed as a race apart, there is perhaps no distinctive body in our country more entitled to compassion and organised care, than the offspring of those whom the outraged law removes from their position as the heads of families to the social extinction, for the time being, of penal servitude or more temporary imprisonment. The matter is one which at the present time claims especial attention from some ominous circumstances. It is an undoubted fact, patent to all who have the opportunity of studying prison records, that the number of cases judicially dealt with for cruel treatment or neglect of children, as well as the more deadly crimes, too often involved in baby farming, have enormously increased. This, to some extent, may be considered due to the salutary work of the Society for the Prevention of Cruelty to Children, which has brought many cases to light that would otherwise have escaped legal notice altogether. Other circumstances have drawn public attention to the nefarious system of baby farming, which in its worst aspect is simply a systematic arrangement for the murder of infants for whom money has been given, on the condition of a solemn pledge that they would be brought up at least in full receipt of the necessities of life. Many of the infants thus handed over to secret and hypocritical assassins are the children of criminals whose incarceration renders their own care of them impossible. Although the representatives of the law have of late years dealt with commendable decision and energy on recognised baby farmers, the iniquitous system is by no means extinct, and we dare scarcely hope that it has been even practically diminished. A recent case affords an unusually emphatic proof of this fact. The daughter of a notorious baby farmer, who was executed not long since for the murder of children committed to her care, has carried on the same profession since her mother's death on the gallows, and is at present in the hands of the police for having hid an infant three weeks old, for whom she had been paid a large sum, under the seat of a railway carriage, where it was not found till the following day. Her manifest purpose in the death of the child was evident from the fact that she had taken away the bottle of milk with which it was being nourished. To rescue the children of criminals from such risks as these, and from others of an equally sinister nature, may be considered the duty of the State; but, pending a recognition of the obligation in that quarter, it seems to call imperatively for the intervention of private benevolence. The system in force at present with regard to these unfortunate waifs is as follows: When a widow is committed to prison, or a married couple, or a man without a lawful wife, the children of such persons are at once taken to the nearest workhouse, and there they remain during the term of their parents' imprisonment; but on the morning of the day when the period of that sentence expires the children are brought from the Union to the prison gate, and there are delivered over to the discharged criminals, and abandoned to whatever fate may be reserved for them in those unworthy hands. Generally speaking, these released prisoners are practically destitute. They may have earned a small gratuity by their work in the prison, or the officials and visitors may have assisted them, and some discharged prisoners' aid society may have helped them for a time; but all such means of support are entirely temporary, and many do not even obtain such assistance at all. As these criminals are not usually persons of high principle or refined sentiment, it is not unnatural when they find themselves with a family of hungry children on their idle hands that they should try to rid themselves of the unwelcome burden as best they can. Parental affection does not hold a very secure place in their moral constitution, and, after all, their predicament is one of serious difficulty. We have ourselves seen nine children brought from the workhouse to the prison to be delivered over to a man and woman who had been hidden away there for six months. We need not descant on the various methods by which these innocent victims of crime may be disposed of, or the existence, morally and socially, to which they are certainly doomed if they are allowed to live.

Let us turn to the more genial theme of suggesting a remedy. We believe that there is but one, albeit somewhat costly, which would fully compass all their sore necessities. An institution ought to be founded to which all the children of criminals should be removed on the conviction of their parents, and there be sheltered, nurtured, and educated so as to become worthy and useful citizens in their native land. It may be said that there are already numberless orphanages, and Homes, and schools to which they might be sent, but for many reasons these do not meet the want. In the first place each separate family or individual child would then require some generous protector to seek out a home for them, and take all the trouble necessary to obtain an entrance to it. Such protectors could only be found for isolated cases, and we desire to see an institution established that would regularly and systematically shelter the whole body of these unoffending pariahs. Also, in many existing Homes, children, whose parents had figured in the police-courts, would not be received, and even if they were would suffer a species of ostracism at the hands of their more happily-born companions. Some time ago a home was found with the greatest difficulty for a little girl, whose father's crime had been of so grave a nature that he paid the penalty with his life. She was only received on a promise being obtained from her that she would never mention the circumstances of her orphanhood. It is not surprising that about a week after her admission, having found a bosom friend in another small child, she confided to her the facts of her history. Instantly she was expelled from the home, and flung back on the hands of the prison visitor who had taken an interest in her case. Is it too much to hope in these days of abounding charity and care for their poorer brethren on the part of persons highly placed and with ample means, that such an institution might be founded for the children of criminals? Although it is at present but a castle in the air, we can see very distinctly how it might be suitably arranged. With two wings, one for boys, the other for girls, and a centre piece fitted as a nursery for the small infants of both sexes, the necessary staff of officials, managers, teachers, and nurses would not be difficult to find. Should this idea commend itself to any willing to aid in such a plan, a letter, addressed X Y Z, care of the Editor of The Hospital, will receive immediate attention.

